# The effect of question order on outcomes in the orbital core outcome set for alcohol brief interventions among online help-seekers (QOBCOS): Findings from a randomised factorial trial

**DOI:** 10.1177/20552076231155684

**Published:** 2023-02-12

**Authors:** Marcus Bendtsen, Claire Garnett, Paul Toner, Gillian W Shorter

**Affiliations:** 1 Department of Health, Medicine and Caring Sciences, 4566 Linköping University, Linköping, Sweden; 2 Department of Behavioural Science and Health, 4919 University College London, London, UK; 3 Centre for Improving Health Related Quality of Life, School of Psychology, 1596 Queen's University Belfast, Belfast, UK; 4 Drug and Alcohol Research Network, Queen's University Belfast, Belfast, UK

**Keywords:** alcohol < lifestyle, trials < studies, public health < disease, epidemiology < medicine, digital < general

## Abstract

**Objective:**

A core outcome set (COS) has been developed in alcohol brief intervention (ABI) research through international consensus. This study aimed to estimate order effects among questions in the COS.

**Methods:**

Individuals aged 18 or older who searched online for alcohol-related help were invited to complete the COS. The order of questions was randomised following a factorial design. Primary outcomes were order effects among the COS items and patterns of attrition.

**Results:**

Between 21/10/2020 and 26/11/2020, we randomised 7334 participants, of which 5256 responded to at least one question and were available for analyses. Current non-drinkers were excluded. We found evidence of higher self-reported average consumption and odds of harmful and hazardous drinking was found among those who first answered questions on recent consumption and impact of alcohol use. Lower self-reported recent consumption was found among those first asked about average consumption. Quality of life (QoL) was reported lower among those who first responded to when questions on impact of alcohol use were asked first, which in turn was lower among those who first answered question on when average consumption and QoL were asked first. Attrition was lowest when average consumption was asked first, and highest when QoL or impact of alcohol use was asked first. Median completion time for the COS was 4.3 min.

**Conclusions:**

Question order affects outcomes and attrition. If the aim is to minimize attrition, consumption measures should be asked before QoL and impact of alcohol use; however, this order impacts self-reported alcohol consumption and so researchers should be guided by study priorities. At a minimum, all participants should be asked the same questions in the same order.

**Trial registration:**

The trial was prospectively registered (ISRCTN17954645).

## Introduction

The World Health Organisation (WHO) has defined alcohol brief interventions (ABIs) as "practices that aim to identify a real or potential alcohol problem and motivate an individual to do something about it".^1^ ABIs aim to help individuals change their behaviour, assess and provide feedback on alcohol use, and motivate and facilitate behaviour change.^2,3^ Over the past 60 years, both face-to-face^1,4,5^ and digital^6–9^ ABIs have been researched and implemented in a wide range of populations, including primary care patients,^5^ emergency health care populations,^10,11^ college students,^12,13^ and veterans.^14^

Comparisons across trials of ABIs, and evidence synthesis of outcomes, are limited because of the high variety of outcome measures used, despite interventions being similar. To overcome this issue, the ORBITAL (Outcome Reporting in Brief Intervention Trials: Alcohol) project was established with the overarching goal of determining an international, consensus-derived, core outcome set (COS).^15^ The aim was to prioritise the key outcomes to be measured in all online, digital, and otherwise delivered ABIs designed for adult drinkers who are at risk or currently experiencing harm but who are not currently in treatment.

The COS used the COMET (Core Outcome Measures in Effectiveness Trials) methodology,^16^ including a systematic review that quantified the diversity in outcomes. In 405 trials of ABIs, they found 2641 different outcomes were used, measured in 1560 different ways.^17^ Following two e-Delphi rounds, a consensus meeting, and psychometric evaluation,^18^ 10 outcomes formed the consensus-derived COS.^19^ The outcomes are:

1. Frequency of drinking
2. Typical number of drinks consumed on a drinking day
3. Frequency of heavy episodic drinking
4. Combined consumption measure
5. Hazardous and harmful drinking
6. Standard drinks consumed in the past week
7. Quality of life
8. Alcohol-related consequences
9. Alcohol-related injury
10. Use of emergency health care services

A consensus was also formed within the ORBITAL project on measures for the COS outcomes. These are listed in Appendix A,^19^ and described in brief here. The WHO's Alcohol Use Disorders Identification Test – Consumption (AUDIT-C) tool^20^ is used to measure the first five outcomes of the COS. There are three questions with scores ranging from 0–4 on each question, and the total score ranging from 0–12 to form the combined consumption measure. A cut-off point of 5+ was used to illustrate hazardous or harmful drinking. The sixth outcome is measured by asking how many standard drinks were consumed each day of the past week, presented as seven questions, one for each day of the week (reported in grams to allow for intercountry comparison). The seventh outcome is measured using the PROMIS (Patient-Reported Outcomes Measurement Information System) global health 1.2 items, a 10 item questionnaire with higher scores indicating higher quality of life.^21^ The eighth outcome is measured using the 15-item Short Index of Problems (SIP) questionnaire.^22,23^ Each item is scored from 0–3 with a 3-month reference period, with scores ranging from 0–45. An additional question based on the SIP regarding injuries inflicted while drinking or being intoxicated (the ninth outcome) was also scored from 0–3. The tenth outcome is measured using a single question about the number of visits to emergency health care services adapted from EconForm90.^24^

This paper reports the Question Order Bias Core Outcome Set (QOBCOS) study, which aimed to assess if there are question order effects among the outcomes of the COS. This type of effect, which can be viewed as a source of bias in measurement, is a well-known phenomenon in marketing and political science.^25,26^ However, more recently, it was suggested that question order effects may exist when measuring alcohol consumption. In exploratory analyses of data collected in a trial, individuals asked to first report weekly alcohol consumption were less likely to be screened as risky drinkers using an average consumption measure, in comparison to those who were first screened using an average consumption measure and then asked about alcohol consumption.^27^ However, in a trial aiming to estimate social desirability bias by randomising order to questions about alcohol dependence and problems and reports on alcohol consumption, no evidence was found that earlier questions biased subsequent reports on alcohol consumption.^28^ The QOBCOS study was conducted to investigate this phenomenon and how it may be a potential source of bias. In addition, we aimed to study patterns of abandonment of the questionnaire to inform how to reduce attrition.

## Methods

A double-blind randomised factorial design trial was used to investigate question order bias among the outcomes of the COS for ABIs. The trial was prospectively registered (ISRCTN Registry ISRCTN17954645) and received ethical approval on 2020-07-01 from the Swedish Ethical Review Authority (Dnr 2020-01799). A trial protocol was also pre-registered.^29^ This report follows the guidelines set out in the CONSORT statement.^30^

### Settings and participants

Individuals searching online for information in the English language on how to drink less or quit drinking were recruited using Google Ads. Examples of search queries targeted were “How do I drink less,” “I drink too much,” and “Support for drinkers”. The adverts were framed as an invitation to take part in a study to improve alcohol intervention research. Individuals who clicked on the advert were asked to read the study information, confirm that they were at least 18 years old and consent to take part. No demographic data on participants was collected to minimise participant burden. This design meant that there was a very low threshold for participation, which, coupled with no incentive for taking part in the study, does increase the risk of high attrition rates.

There were no explicit exclusion criteria; however, as decided a priori,^29^ analyses excluded those who reported having not consumed any alcohol during the past three months (i.e., answering *Never* to the to the first AUDIT-C question and having consumed zero drinks in the past week).

### Interventions

The 10 COS outcomes were divided into four clusters: (1) **average consumption**: frequency of drinking, typical number of drinks consumed on a drinking day, frequency of heavy episodic drinking, combined summary consumption measure, hazardous and harmful drinking; (2) **recent consumption**: standard drinks consumed in the past week; (3) **quality of life**: health-related quality of life; and (4) **impact of alcohol use**: alcohol-related consequences, alcohol-related injury, use of emergency health care services. The order of the four clusters was permuted to create 24 (=4!) order combinations, i.e., 24 conditions to which participants were randomised. Table 1 shows the conditions and different permutations to which participants were allocated.

**Table 1.** Order combinations of the four item clusters creating 24 trial conditions.

Condition	Presentation order of clusters			
**1**	1	2	3	4
**2**	1	2	4	3
**3**	1	3	2	4
**4**	1	3	4	2
**5**	1	4	2	3
**6**	1	4	3	2
**7**	2	1	3	4
**8**	2	1	4	3
**9**	2	3	1	4
**10**	2	3	4	1
**11**	2	4	1	3
**12**	2	4	3	1
**13**	3	1	2	4
**14**	3	1	4	2
**15**	3	2	1	4
**16**	3	2	4	1
**17**	3	4	1	2
**18**	3	4	2	1
**19**	4	1	2	3
**20**	4	1	3	2
**21**	4	2	1	3
**22**	4	2	3	1
**23**	4	3	1	2
**24**	4	3	2	1

Note: **1** = Average consumption; **2** = Recent consumption; **3** = Quality of life; **4** = Impact of alcohol use

Questions were presented to participants in the order corresponding to their allocation. All questions were shown on the same page, with the next question revealed after responding to the current question. To make the trial similar to regular surveys, participants were allowed to go back and change their responses to previous questions. Once all questions had been answered, participants were thanked and recommended to read more about alcohol and health on a selection of websites (listed in Appendix B). Contact information for the primary investigator was provided to participants as part of informed consent materials, however, there was no direct interaction between the study team and participants.

### Outcomes

The primary outcomes were: (i) the 10 outcomes of the COS measured using the recommended questionnaires (listed in Appendix A^19^), and (ii) the proportion of participants abandoning the questionnaire. These outcomes facilitated the primary analysis of order effects. Since the COS is new, the abandonment rate can guide future trials adopting the COS.

There were two secondary outcomes: (i) time spent on the questionnaire among completers and abandoners to estimate the anticipated burden of completing the COS, and (ii) the proportion of participants visiting the links provided at the end of the questionnaire to show if responding to the COS satisfied participants’ intentions to seek help online, and if this differed by response to the COS. Analyses of the proportions visiting the provided links are reported separately.

### Sample size

The trial used a Bayesian group sequential design^31–33^; thus, a set of target criteria were evaluated continuously to decide when recruitment would end. The primary analyses were repeated periodically as data were collected and the posterior distributions of coefficients representing cluster order effects were assessed for evidence of effect or futility.

Let ß_k,i_ represent the coefficients for each order effect (*i* = 1,2,3) in each model (*k* = 1…10) and D represent the data available at the interim analyses. Then, the target criteria were:

- **Effect**: p(ß_k,i_ > 0 | D) > 97.5% or p(ß_k,i_ < 0 | D) > 97.5% (i.e., if the question order effect was greater or less than the null with a probability greater than 97.5%)
- **Futility** (linear regression): p(−0.1 < ß_k,i_ < 0.1 | D) > 95% (i.e., if the question order effect is close to the null with a probability greater than 95%)
- **Futility** (negative binomial and logistic regression): p(log(1/1.2) < ß_k,i_ < log(1.2)) > 95% (i.e., if the question order effect is close to the null with a probability greater than 95%)

For the effect criterion, a sceptical normal prior was used for regression coefficients (mean=0, SD=1.0), and a wider prior was used for the futility criteria (mean=0, SD=2.0).

As is the case in Bayesian adaptive designs, these criteria were used as guides to aid the decision of when to stop recruitment, rather than strict a priori defined rules.^31–33^ By virtue of sceptical priors, estimates are pulled towards the null when data is scarce, protecting against spurious and potentially erroneous findings. Due to the nature of Bayesian inference, there is no need to adjust analyses for multiple looks at the data.^34^

### Randomisation

Block randomisation was used to achieve equal allocation among arms (random block sizes of 24 and 48 were used to ensure that the sequence could not be predicted). The randomisation sequence and allocation were fully automated and computerised, leaving researchers blinded throughout the study period. Participants were aware they were taking part in a research study; however, the true nature of the study was not revealed to them, since this would interfere with the effects being studied. Therefore, participants were also blinded to their allocation.

Since no identifiers were collected for individuals, we used web-browser cookies and HTML5 storage to store allocation information on the participants’ web-browsers. Participants who had not completed the questionnaire and returned to the trial website were presented with the cluster order according to their assignment. Participants who had completed the questionnaire and returned to the trial website were thanked for their participation, but not offered an opportunity to answer the questions again.

### Analysis

All analyses were conducted following intention-to-treat principles, with all participants analysed in the groups to which they were randomised. As was pre-specified, current non-drinkers, identified by responding *Never* to the first AUDIT-C item and having not consumed any alcohol the past week, were excluded from analyses. Since causal mechanisms leading to missing data in this study were unknown, and it was anticipated attrition would be high, complete case analyses were planned as primary, with sensitivity analyses conducted with imputed data (multiple imputation with chained equations). Imputation was done using responses to all questions in the questionnaire. Imputed analyses included participants who responded to at least one question; thus, excluding participants for which no data at all was available. Non-drinkers were included to improve multiple imputation but were subsequently removed from imputed analyses, as were individuals with imputed values suggesting that they were current non-drinkers.

Estimates of model parameters were interpreted by inspecting marginal posterior distributions using Bayesian inference^35^ (see Sample Size for specifications of priors). The Bayesian analysis treats evidence as a continuous measure, where it is the relative compatibility between different parameter values and the data that is studied. Thus, there is no decision whether there is or is no evidence for a studied phenomenon. We complemented this analysis with null hypothesis testing, which treats evidence as dichotomous, and estimates the probability of the data given that the parameter value is fixed at the null. To reject the assumption that the parameter value was null, we used the conventional alpha level of 0.05. These two approaches each give their own side of the story, one where data is fixed, and parameters are not, and vice versa. Both approaches were used for our scientific inference, thus evidence is considered both continuous and dichotomous in this analysis.

#### Primary analyses

The primary analysis of question order effects was conducted by using regression models in which each outcome in each cluster was regressed against a dummy variable representing whether each of the other clusters was asked before or after the outcome. For instance, standard drinks consumed in the past week (Cluster 2), was regressed against three dummy variables, representing Cluster 1, Cluster 3, and Cluster 4, respectively. The dummy variables took value 0 if the cluster was asked after Cluster 2 and value 1 if the cluster was asked before Cluster 2. For each outcome, one regression model was estimated, yielding a total of 10 models, using negative binomial regression for counts (past week's consumption and number of visits to emergency health care services), logistic regression for hazardous or harmful drinking (using AUDIT-C scores of 5+ as cut-off), and normal regression for scores (all other outcomes). We investigated 2- and 3-way interactions among cluster dummy variables to explore if the order of a combination of clusters affects outcomes.

The proportion of participants abandoning the questionnaire was analysed in two ways. First, to identify cluster orders that were more likely to result in abandoning the questionnaire, logistic regression was used to model abandonment versus completion with allocated arm as a covariate. Second, to identify clusters more likely to result in abandonment, multinomial regression was used to model the abandoned cluster (i.e., the cluster which was being presented when participants abandoned the survey). To account for the different number of questions within each cluster, the model of abandoned cluster was adjusted for the number of questions responded to. Both models were conducted using standard normal priors.

#### Secondary analyses

Time spent on the questionnaire was analysed in two ways. First, using normal regression with allocation as a covariate among both completers and abandoners. Second, using normal regression with the COS outcomes as covariates (completers only). Both analyses were conducted under standard normal priors, and the second analysis was also conducted using shrinkage priors.

## Results

A total of 7334 participants were randomised from 21^st^ October and 26^th^ November 2020. At this time, the a priori defined target criteria (see Sample Size) were found to be sufficiently fulfilled that a decision was made to stop recruitment. See Appendix C for the final evaluation of the target criteria. Among randomised participants, 2078 did not respond to any questions at all. There were 475 participants who were current non-drinkers, leaving 4781 participants with partial responses who could be included in analyses of outcome measures where data was available. Imputation analyses were performed among the 5256 who had responded to at least one question, with non-drinkers removed before analysis.

Descriptive data of the study population are presented in Table 2, using available data for each outcome, excluding current non-drinkers. Approximately 61% of those included were classified as hazardous or harmful drinkers using average consumption measures. Past week's drinking was considerable, with mean consumption at 322 grams of alcohol (SD=298). The impact of alcohol use was reflected by a mean score on the Short Index of Problems (SIP) at 13.7 (SD = 11.5). There was some recent use of emergency healthcare reported, with a low proportion of injury.

**Table 2.** Descriptive data of the analysed study population (excluding current non-drinkers).

	**Mean (SD)^a^**	**n (%)^a^**
AUDIT-C Item 1	2.55 (1.44)	
AUDIT-C Item 2	1.39 (1.37)	
AUDIT-C Item 3	1.78 (1.44)	
AUDIT-C Total	5.72 (3.66)	
Past week consumption (grams)	322 (298)	
PROMIS Global 10	32.8 (8.2)	
Short Index of Problems	13.7 (11.5)	
Injury	0.58 (0.98)	
Emergency health care services	1.19 (3.20)	
Hazardous and harmful drinking		2170 (61.2)

^a^ Among participants for whom data was available.

### Primary analyses

#### AUDIT-C items, total score, and hazardous and harmful drinking - cluster 1

Estimates of order effects on outcomes within Cluster 1 (average consumption) are shown in Table 3.

**Table 3.** Estimates of order effects of Cluster 1, 2, and 4 on AUDIT-C items, total score, and hazardous and harmful drinking.

	Cluster 2 recent consumption (Past week consumption)		Cluster 3 quality of life (PROMIS Global 10)		Cluster 4 impact of alcohol use (SIP + Injury + Emergency)	
	Post. median and 95% CI ^a^	Probability of effect ^b^ P-value ^c^	Post. median and 95% CI ^a^	Probability of effect ^b^ P-value^c^	Post. median and 95% CI ^a^	Probability of effect ^b^ P-value^c^
**AUDIT-C Total (linear coefficients)**						
Complete case (n = 3544)	0.26 (−0.01;0.53)	97.0% 0.059	−0.31 (−0.58; −0.04)	98.8% 0.020	0.69 (0.42; 0.96)	> 99.9% < 0.001
Imputed	0.23 (−0.03; 0.49)	96.0% 0.080	−0.34 (−0.59; −0.09)	99.6% 0.008	0.35 (0.09; 0.60)	99.7% 0.007
**AUDIT 1 (linear coefficients)**						
Complete case (*n* = 3652)	0.16 (0.05; 0.26)	99.9% 0.003	−0.10 (−0.20; 0.01)	96.1% 0.079	0.26 (0.16;0.37)	> 99.9% < 0.001
Imputed	0.11 (0.01; 0.22)	98.4% 0.032	−0.11 (−0.21; −0.01)	98.7% 0.027	0.14 (0.04; 0.24)	99.6% 0.008
**AUDIT 2 (linear coefficients)**						
Complete case (*n* = 3585)	0.01 (−0.09; 0.11)	57.2% 0.85	−0.12 (−0.22; −0.01)	98.7% 0.025	0.26 (0.16; 0.36)	> 99.9% < 0.001
Imputed	0.03 (−0.06; 0.13)	74.9% 0.50	−0.11 (−0.21; −0.01)	98.7% 0.027	0.15 (0.05; 0.24)	99.9% 0.003
**AUDIT 3 (linear coefficients)**						
Complete case (*n* = 3552)	0.09 (−0.01;0.20)	95.7% 0.086	−0.12 (−0.22; −0.01)	98.3% 0.033	0.18 (0.07;0.29)	> 99.9% < 0.001
Imputed	0.09 (−0.02; 0.19)	95.0% 0.099	−0.12 (−0.22; −0.02)	99.2% 0.016	0.07 (−0.03; 0.17)	90.5% 0.20
**Hazardous and harmful drinking (AUDIT-C Total >= 5) (odds ratios)**						
Complete case (*n* = 3544)	1.13 (0.97; 1.31)	93.5% 0.13	0.95 (0.81; 1.10)	76.2% 0.47	1.33 (1.14;1.55)	> 99.9% < 0.001
Imputed	1.11 (0.96; 1.29)	92.5% 0.15	0.90 (0.78; 1.04)	92.5% 0.15	1.14 (0.99; 1.31)	96.4% 0.074

^a^ The point estimate in the table is the median of the marginal posterior distribution for the regression coefficient representing if the cluster was asked before (vs after) the outcome. For AUDIT-C 1, 2, 3 and Total, the coefficient represents the difference in outcome scores if the cluster was asked before (vs after) the outcome. For hazardous and harmful drinking, the coefficient represents the odds ratio when asking the cluster before (vs after) the outcome. The 95% compatibility interval is represented by the 2.5% and 97.5% quartiles of the marginal posterior distributions.

^b^ The proportion of the marginal posterior distribution which is in the same direction as the median below/above the null (0 for linear coefficients and 1 for odds ratios).

^c^ The *P*-value was calculated based on the maximum likelihood estimate of regression coefficients. These are not displayed in the table as almost identical to the posterior median in this large sample.

There was evidence that the total AUDIT-C score was higher among those first asked about their past week alcohol consumption (beta = 0.26; 95% CI = −0.01; 0.52; probability of effect 97.0%; *P*-value=0.059), largely driven by differences in responses to the first and third AUDIT-C items. The odds of being classified as a hazardous or harmful drinker was also higher among those first asked about past week consumption, albeit with weaker evidence (OR = 1.13, 95% CI = 0.97; 1.31, probability of effect 93.5%; P-value = 0.13).

Conversely, there was evidence that first being asked the PROMIS Global 10 questionnaire (Cluster 3) resulted in lower total AUDIT-C scores (beta = −0.31; 95% CI = −0.58; −0.04; probability of effect = 98.8%; P-value = 0.020), with evidence that responses to all three AUDIT-C items were affected in the same direction. There was however no marked difference in odds of hazardous and harmful drinking from responding to PROMIS Global 10 first.

Finally, AUDIT-C scores were higher among those who had first responded to the SIP, injury, and emergency questions (Cluster 4) (beta = 0.69; 95% CI = 0.42; 0.96; probability of effect > 99.9%; *P*-value < 0.001). All three AUDIT-C items were higher among those first responding to the questions in Cluster 4. Odds of hazardous or harmful drinking was also higher amongst those who first asked the questions in Cluster 4 (OR = 1.33; 95% CI = 1.14; 1.54; probability of effect > 99.9%; *P*-value < 0.001). Notably, the estimated order effects of Cluster 4 on Cluster 1 were markedly attenuated in the imputed analyses, thus, these may be taken as a more conservative estimate of order effects.

There was no evidence of any marked interaction effects with the order of cluster 2, 3, and 4 on any of the outcomes in the first cluster.

#### Past week consumption – cluster 2

Estimate of order effects on cluster 2 (recent consumption) are shown in Table 4.

**Table 4.** Estimates of order effects of Cluster 1, 3 and 4 on recent consumption.

	Cluster 1 average consumption (AUDIT-C)		Cluster 3 quality of life (PROMIS Global 10)		Cluster 4 impact of alcohol use (SIP + Injury + Emergency)	
	Post. median and 95% CI ^a^	Probability of effect ^b^ *P*-value ^c^	Post. median and 95% CI ^a^	Probability of effect ^b^ *P*-value^c^	Post. median and 95% CI ^a^	Probability of effect ^b^ *P*-value^c^
**Past week consumption (grams) (incidence rate ratios)**						
Complete case (n = 3408)	0.91 (0.84; 0.99)	98.7% 0.023	1.00 (0.93; 1.08)	52.7% 0.97	1.00 (0.92; 1.08)	54.1% 0.91
Imputed	0.92 (0.85; 0.99)	99.0% 0.013	0.98 (0.91; 1.06)	69.0% 0.59	0.97 (0.90; 1.05)	76.1% 0.43

^a^ The point estimate in the table is the median of the marginal posterior distribution for the regression coefficient representing if the cluster was asked before (vs after) the outcome. The coefficient represents the incidence rate ratio when asking the cluster before (vs after) the outcome. The 95% compatibility interval is represented by the 2.5% and 97.5% quartiles of the marginal posterior distributions.

^b^ The proportion of the marginal posterior distribution which is in the same direction as the median below/above the null (1 for incidence rate ratios).

^c^ The P-value was calculated based on the maximum likelihood estimate of regression coefficients. These are not displayed in the table as almost identical to the posterior median in this large sample.

The evidence suggested that those who responded to the AUDIT-C questionnaire (cluster 1) before past week consumption reported 9% fewer grams of alcohol than those responding to AUDIT-C after (IRR = 0.91, 95% CI = 0.84; 0.99, probability of effect 98.7%; *P*-value = 0.023). There was no evidence of any marked order effect from Cluster 3 or Cluster 4 on past week consumption, nor any interaction effects with the order of Clusters 1, 3, and 4.

#### PROMIS global 10 – cluster 3

Estimates of order effects on Cluster 3 (quality of life) are shown in Table 5.

**Table 5.** Estimates of order effects of Cluster 1, 2 and 4 on PROMIS Global 10 scores.

	Cluster 1 average consumption (AUDIT-C)		Cluster 2 recent consumption (Past week consumption)		Cluster 4 impact of alcohol use (SIP + Injury + Emergency)	
	Post. median and 95% CI ^a^	Probability of effect ^b^ *P*-value ^c^	Post. median and 95% CI ^a^	Probability of effect ^b^ *P*-value^c^	Post. median and 95% CI ^a^	Probability of effect ^b^ *P*-value^c^
**PROMIS Global 10 (linear coefficients)**						
Complete case (*n* = 3369)	−0.26 (−0.84; 0.30)	82.3% 0.37	−0.09 (−0.66; 0.48)	62.8% 0.79	−0.98 (−1.56; −0.41)	> 99.9% < 0.001
Imputed	−0.20 (−0.74; 0.35)	76.2% 0.50	−0.33 (−0.89; 0.22)	88.2% 0.25	−0.71 (−1.27; −0.16)	99.4% 0.012

^a^ The point estimate in the table is the median of the marginal posterior distribution for the regression coefficient representing if the cluster was asked before (vs after) the outcome. The coefficient represents the difference in outcome scores if the cluster was asked before (vs after) the outcome. The 95% compatibility interval is represented by the 2.5% and 97.5% quartiles of the marginal posterior distributions.

^b^ The proportion of the marginal posterior distribution which is in the same direction as the median below/above the null (0 for linear coefficients).

^c^ The *P*-value was calculated based on the maximum likelihood estimate of regression coefficients. These are not displayed in the table as almost identical to the posterior median in this large sample.

There was no evidence of any marked order effects with Cluster 1 and Cluster 2. There was however evidence that those responding to the SIP, injury, and emergency questions (Cluster 4) before the PROMIS Global 10 questions reported lower quality of life scores (beta = −0.98; 95% CI = −1.56; −0.41; probability of effect > 99.9%; *P*-value < 0.001). No interaction effects with the order of Cluster 1, 2, and 4 on PROMIS Global 10 were observed.

#### Short inventory of problems, injury, and emergency health care visits – cluster 4

Estimates of order effects on Cluster 4 (impact of alcohol use) are shown in Table 6.

**Table 6.** Estimates of order effects of Cluster 1, 2, and 3 on Short Inventory of Problems scores, injury, and emergency health care visits.

	Cluster 1 average consumption (AUDIT-C)		Cluster 2 recent consumption (Past week consumption)		Cluster 3 quality of life (PROMIS Global 10)	
	Post. median and 95% CI ^a^	Probability of effect ^b^ *P*-value ^c^	Post. median and 95% CI ^a^	Probability of effect ^b^ *P*-value^c^	Post. median and 95% CI ^a^	Probability of effect ^b^ *P*-value^c^
**SIP (linear coefficients)**						
Complete case (*n* = 3287)	−1.14 (−1.91; −0.40)	99.8% 0.003	−0.32 (−1.07; 0.44)	79.2% 0.51	−1.13 (−1.87; −0.37)	99.8% 0.004
Imputed	−1.02 (−1.75; −0.28)	99.6% 0.006	0.17 (−0.55; 0.89)	67.4% 0.54	−1.04 (−1.77; −0.33)	99.8% 0.004
**Injury (linear coefficients)**						
Complete case (*n* = 3244)	−0.04 (−0.11; 0.03)	88.0% 0.24	−0.02 (−0.10;0.05)	73.2% 0.54	−0.05 (−0.12;0.03)	87.8% 0.24
Imputed	−0.03 (−0.10;0.03)	83.7% 0.33	0.01 (−0.06; 0.08)	61.0% 0.78	−0.05 (−0.12; 0.02)	90.2% 0.20
**Emergency health care visits (incidence rate ratios)**						
Complete case (*n* = 3215)	0.97 (0.76; 1.22)	61.4% 0.78	0.88 (0.69; 1.11)	87.4% 0.28	1.07 (0.84; 1.36)	70.6% 0.59
Imputed	1.00 (0.81; 1.23)	51.5% 0.98	1.03 (0.84; 1.27)	61.4% 0.75	1.05 (0.85; 1.29)	66.1% 0.65

^a^ The point estimate in the table is the median of the marginal posterior distribution for the regression coefficient representing if the cluster was asked before (vs after) the outcome. For SIP and Injury, the coefficient represents the difference in outcome scores if the cluster was asked before (vs after) the outcome. For emergency health care visits, the coefficient represents the incidence rate ratios when asking the cluster before (vs after) the outcome. The 95% compatibility interval is represented by the 2.5% and 97.5% quartiles of the marginal posterior distributions.

^b^ The proportion of the marginal posterior distribution which is in the same direction as the median below/above the null (0 for linear coefficients and 1 for incidence rate ratios).

^c^ The *P*-value was calculated based on the maximum likelihood estimate of regression coefficients. These are not displayed in the table as almost identical to the posterior median in this large sample.

There was evidence of lower SIP scores among those who had responded to the AUDIT-C questionnaire (Cluster 1) prior to SIP (beta = −1.14; 95% CI = −1.91; −0.40; probability of effect 99.8%; P-value = 0.003), as well as those who had responded to PROMIS Global 10 before SIP (beta = −1.13; 95% CI = −1.87; −0.37; probability of effect 99.8%; P-value = 0.004). There was no evidence suggesting any marked order effect of Cluster 2 on SIP scores.

The evidence suggested no order effects from any of the clusters on the questions relating to injury and emergency health care visits. Also, no interaction effects with the order of Clusters 1, 2, and 3 were observed on any of the outcomes in the fourth cluster.

#### Abandonment

**Figure 1.**
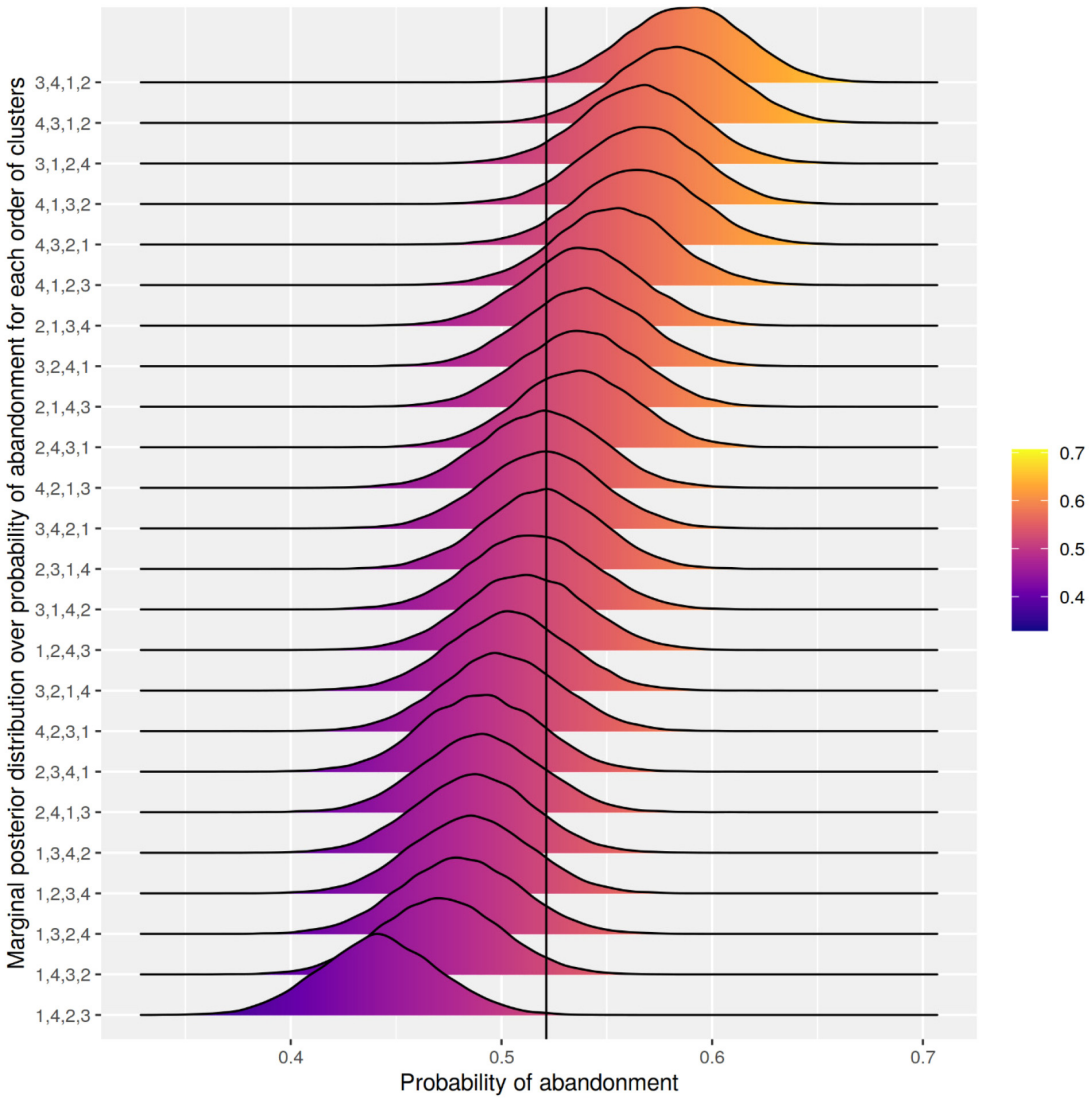
Marginal posterior distribution over the probability of abandoning the questionnaire in each of the 24 conditions. Note: 1 = Average consumption; 2 = Recent consumption; 3 = Quality of life; 4 = Impact of alcohol use.

Figure 1 illustrates the marginal posterior distributions over the probability of abandoning the questionnaire in each of the 24 conditions (including all 7334 randomised participants). The mean abandonment rate was 52%, illustrated by the vertical line in Figure 1. Conditions which started with alcohol consumption measures (clusters 1 and 2) were less likely to be abandoned than conditions that started with questions relating to quality of life and the impact of alcohol use (clusters 3 and 4).

Among those who abandoned the questionnaire, there were more participants doing so while responding to Cluster 2, 3 and 4 in comparison with Cluster 1 (AUDIT-C items), adjusted for the number of questions responded to. The median posterior odds ratio for abandoning Cluster 2 vs Cluster 1 was 1.45 (95% CI = 1.29; 1.63, probability of association > 99.9%); Cluster 3 vs Cluster 1 was 1.47 (95% CI = 1.31; 1.64, probability of association > 99.9%); and Cluster 4 vs Cluster 1 was 1.71 (95% CI = 1.54; 1.91, probability of association > 99.9%).

### Secondary analyses

Participants spent a median of 100 s responding to the questionnaire (IQR: 0; 256), including the 2078 participants who did not respond to any questions at all (for which the time spent was 0 s). Among those who completed at least one question, the median time spent was 200.5 s (IQR: 71; 298). Among completers, the median time spent was 256 s (IQR: 190; 348). There was no consistent evidence suggesting the order of clusters was associated with overall time spent on the questionnaire among both abandoners and completers. Similarly, among completers, there were no marked differences in time spent on the questionnaire regarding responses to the COS.

## Discussion

We employed a factorial randomised trial to estimate order effects among the outcomes of the COS for ABIs, and to investigate patterns of abandonment. We found evidence that first responding to questions about average consumption led to subsequent lower self-report of recent consumption, and lower impact of alcohol use. First responding to questions about recent consumption, however, led to higher scores on average consumption. We also found evidence that first responding to questions regarding quality of life led to lower self-report of average consumption and lower impact of alcohol use. Finally, first responding to questions regarding impact of alcohol use led to higher average consumption, including being more likely to be classified as a hazardous/harmful drinker, and lower quality of life.

### Interpretation of findings

The causal mechanisms leading to the order effects identified in this study are unknown and were not explicitly measured. However, potential reasons for the effects include using one's own answers to earlier questions to inform answers to subsequent questions,^36^ perhaps altering responses to be consistent across questions. Also, reflecting on the impact of alcohol use may also be a potential reason why recall of alcohol consumption was affected. For instance, having first responded to average consumption measures, participants may have altered their report of recent consumption to be closer to that of an average week's consumption. Since participants were recruited while searching for help online to reduce their drinking, they may have recently felt that they drank too much or experienced an adverse event and as such their average weekly consumption may be less than recent weekly consumption. Conversely, having first reported recent consumption, participants may have reminded themselves of what an average week's consumption looks like, or attempted to be more consistent in their responses, and thus reported higher average weekly consumption than if not having this opportunity to reflect.

Apart from order effects, this trial also identified a pattern of abandonment suggesting that first being asked about alcohol consumption measures resulted in less abandonment than first being asked about quality of life or impact of alcohol use. Abandonment was more likely while responding to questions regarding recent consumption, quality of life, and impact of alcohol use, in comparison to average consumption (adjusted for number of questions responded to). Previous research has found attrition is higher when participants in alcohol studies are asked to respond to questionnaires perceived to be less relevant to them.^37^ Participants were expecting to answer questions about alcohol and were therefore likely prepared to answer questions about consumption. Being asked questions about quality of life and impact of alcohol may then have resulted in a higher than anticipated cognitive effort, reflection on consumption, emotions about consumption, or feeling judged about their alcohol use.

### Previous research

A previous study has suggested that those who are first asked about recent consumption are later less likely to be screened as hazardous or harmful drinkers using AUDIT-C (OR = 0.83; 95% CI = 0.70–0.99),^27^ however, our findings showed the opposite. The decision to analyse question order effects appeared post-hoc (not mentioned in protocol or trial registration) in an intervention trial. The trial also used AUDIT-C to determine hazardous/harmful drinking although the full AUDIT was collected. In contrast, the current study was purposely designed to study order effects with a pre-registered protocol and statistical analysis plan.^29^

The COS uses the alcohol consumption subscale (AUDIT-C) from the full AUDIT screening tool, which has an additional factor of alcohol dependence and problems.^20,38^ In a study of social desirability bias,^28^ order effects of first responding to the dependence and problems subscales before the AUDIT-C were estimated. The study found no evidence of any marked order effects, which conflicts with our findings. There are several potential reasons why these studies show different results, including that the COS recommends SIP to assess alcohol problems rather than the AUDIT subscale, and the issues of the AUDIT questions 9 and 10 to measure change over time.^19^ The SIP is a dedicated and detailed inventory of alcohol-related problems and may invite more reflection on alcohol consumption.

There may be a number of differences in population composition and drinking intention which could explain the equivocal findings. We targeted individuals with search terms around help with drinking and improving research on alcohol interventions. Other studies are often aiming to develop or evaluate interventions. Similarly, we had 475 individuals sign up who were not current drinkers, which diverges somewhat from intervention research, although we excluded these from our analyses. Participants were also asked if the aim of their participation was to get help to reduce their alcohol use, 29% of responders (1020/3494) said no. Again, this diverges from typical intervention research.

### Implications for research and practice

Some of the effects we observed in this study are small, and so whether these effects are relevant depends on the context in where one expects to observe them. For instance, when comparing brief intervention studies, differences in estimates may be partially due to the way in which questions were asked rather than interventions being different. Perhaps more problematic, if questionnaires were designed differently for intervention and control participants within a trial, the order effects may at least partially mask (or inflate) the observed effect – although, where effects of interventions are large, it may have less relevance and can possibly be disregarded. On the other hand, weekly alcohol consumption was lower by 9% when AUDIT-C was asked first, which could be a substantial part of the effect one expects from some brief interventions.

There appear to be order effects in how the data is presented, and the intent of the investigators may determine in which order questions should be presented. For example, it is widely considered that self-reported alcohol consumption often underestimates the alcohol consumed.^39^ If the intent is to capture a higher self-report, a potential option may be to present impact of alcohol use and recent consumption first, followed by average consumption and quality of life. However, this may have a penalty in relation to retention, where average consumption works best as the first cluster. A trial that aims to include hazardous and harmful drinkers will likely include more participants if asked to respond to the impact of alcohol use measures or recent consumption before average consumption. If used in a screening and brief intervention setting, where the feedback and advice are dynamic based on participant responses, the order in which questions are asked during screening could affect the advice given, which should be borne in mind when designing intervention materials. Finally, if the COS outcomes are used in survey research, findings may be influenced by order effects. Care should be taken when comparing findings from different surveys or registries, and when possible, analyses should account for the uncertainty in outcome measures which order effects may produce.

### Limitations and generalisability

This was an online study that did not require individuals to verify any personal identifiers, rather, we relied on web-browser storage to ensure that individuals were randomised once. However, this means that there is an unknown risk that participants used a different device or web-browser to participate multiple times. Order effects could be reduced once having been exposed to all questions and completing them again, thus, our effect estimates may be biased towards the null. There were 7% (484/7335) of participants who visited the study site multiple times from the same web-browser (visits within the same hour were not counted to differentiate between revisits and reloading of the page), thus, interest in the study site was relatively low after initial contact and there was no financial incentive which could have motivated multiple participation.

Several participants (n = 2078) did not respond to a single question. This is somewhat unsurprising, considering that individuals may simply have been curious about the website or study, and therefore clicked on the advert and consented without much reflection. Faced with questions about alcohol and health may have discouraged further exploration. This does limit our ability to infer unbiased intention-to-treat estimates, and there is no data available for imputation for these individuals. We ran our sensitivity analyses with imputed data among participants who had at least responded to one question, which attenuated some of the effect estimates, but did not change our overall findings.

The COS is designed to be used at follow-up in alcohol intervention studies, thus, participants at baseline have been asked similar questions and been screened into the study. In an intervention study, participants may potentially be protected against question order effects, due to previously responding to similar questions. However, it remains an open question if the order effects identified in this trial are persistent over longer periods of time, or if they only affect immediate subsequent responses. As others have noted, it would be interesting to see the intersection with objective measures such as biomarkers or ecological momentary assessment measures.^39,40^ Finally, we strongly encourage researchers to replicate this study to develop stronger, international evidence on question order effects; those interested in doing so are invited to contact the corresponding author.

## Conclusions

We found evidence of order effects among the four clusters of ORBITAL COS outcomes. Researchers designing studies which include measures of average and recent consumption, quality of life, and the impact of alcohol use should be aware of these effects and design (and pre-register) their studies accordingly. At a minimum, all study participants should be asked the same questions in the same order. Researchers should be guided by the nature of the studied population, recruitment, additional questions, concerns about under-reporting, screening for inclusion, and retention concerns. For instance, if the aim is to reduce attrition, consumption measures should be asked before the quality of life and impact of alcohol use; however, this order affects self-reported consumption and should therefore be balanced with study priorities. Replication for stronger, international evidence for question order effects will better guide researchers in decision making. The COS is practical, can be responded to in reasonable time, with less attrition if average consumption measures are asked first.

## Supplemental Material

sj-docx-1-dhj-10.1177_20552076231155684 - Supplemental material for The effect of question order on outcomes in the orbital core outcome set for alcohol brief interventions among online help-seekers (QOBCOS): Findings from a randomised factorial trial Click here for additional data file. Supplemental material, sj-docx-1-dhj-10.1177_20552076231155684 for The effect of question order on outcomes in the orbital core outcome set for alcohol brief interventions among online help-seekers (QOBCOS): Findings from a randomised factorial trial by Marcus Bendtsen, Claire Garnett, Paul Toner and Gillian W Shorter in Digital Health

sj-docx-2-dhj-10.1177_20552076231155684 - Supplemental material for The effect of question order on outcomes in the orbital core outcome set for alcohol brief interventions among online help-seekers (QOBCOS): Findings from a randomised factorial trial Click here for additional data file. Supplemental material, sj-docx-2-dhj-10.1177_20552076231155684 for The effect of question order on outcomes in the orbital core outcome set for alcohol brief interventions among online help-seekers (QOBCOS): Findings from a randomised factorial trial by Marcus Bendtsen, Claire Garnett, Paul Toner and Gillian W Shorter in Digital Health

sj-docx-3-dhj-10.1177_20552076231155684 - Supplemental material for The effect of question order on outcomes in the orbital core outcome set for alcohol brief interventions among online help-seekers (QOBCOS): Findings from a randomised factorial trial Click here for additional data file. Supplemental material, sj-docx-3-dhj-10.1177_20552076231155684 for The effect of question order on outcomes in the orbital core outcome set for alcohol brief interventions among online help-seekers (QOBCOS): Findings from a randomised factorial trial by Marcus Bendtsen, Claire Garnett, Paul Toner and Gillian W Shorter in Digital Health
