# Supplementary material for: The effect of question order on outcomes in the orbital core outcome set for alcohol brief interventions among online help-seekers (QOBCOS): Findings from a randomised factorial trial
Source: Digit Health. 2023 Feb 12;9:20552076231155684. doi: 10.1177/20552076231155684 (PMC9926362; doi:10.1177/20552076231155684)
Supplement: sj-docx-1-dhj-10.1177_20552076231155684 - Supplemental material for The effect of question order on outcomes in the orbital core outcome set for alcohol brief interventions among online help-seekers (QOBCOS): Findings from a randomised factorial trial [file sj-docx-1-dhj-10.1177_20552076231155684.docx]

# Appendix A – Measures for the COS outcomes

For a full data dictionary, please see: Shorter GW, Bray JW, Heather N, Berman AH, Giles EL, Clarke M, et al. The "Outcome Reporting in Brief Intervention Trials: Alcohol" (ORBITAL) Core Outcome Set: International Consensus on Outcomes to Measure in Efficacy and Effectiveness Trials of Alcohol Brief Interventions. *Journal of Studies on Alcohol and Drugs*. 2021;82(5):638-46.

## Cluster 1

1. In the past 3 months, how often have you had a drink containing alcohol?
   1. Never
   2. Monthly or less
   3. 2-4 times a month
   4. 2-3 times a week
   5. 4 or more times a week
2. In the past 3 months, how many drinks containing alcohol have you had on a typical day when you were drinking?
   1. 1 or 2
   2. 3 or 4
   3. 5 or 6
   4. 7 to 9
   5. 10 or more
3. In the past 3 months, how often did you have six or more drinks on one occasion?
   1. Never
   2. Less than monthly
   3. Monthly
   4. Weekly
   5. Daily or almost daily

Note: A visual guide will be offered with the definition of a standard drink.

## Cluster 2

1. Thinking about the past week, how many standard drinks did you have on:
   1. Monday
   2. Tuesday
   3. Wednesday
   4. Thursday
   5. Friday
   6. Saturday
   7. Sunday

Note: A visual guide will be offered with the definition of a standard drink.

## Cluster 3

Considering the period of the past three months:

1. In general, would you say your health is: (Poor, Fair, Good, Very good, Excellent)
2. In general, would you say your quality of life is: (Poor, Fair, Good, Very good, Excellent)
3. In general, how would you rate your physical health: (Poor, Fair, Good, Very good, Excellent)
4. In general, how would you rate your mental health, including your mood and your ability to think? (Poor, Fair, Good, Very good, Excellent)
5. In general, how would you rate your satisfaction with your social activities and relationships? (Poor, Fair, Good, Very good, Excellent)
6. In general, please rate how well you carry out your usual social activities. This includes activities at home, at work and in your community, and responsibilities as a parent, child, spouse, employee, friend, etc.: (Poor, Fair, Good, Very good, Excellent)
7. To what extent are you able to carry out your everyday physical activities such as walking, climbing stairs, carrying groceries, or moving a chair?
   1. Not at all
   2. A little
   3. Moderately
   4. Mostly
   5. Completely
8. In the past 7 days, how often have you been bothered by emotional problems such as feeling anxious depressed or irritable?
   1. Always
   2. Often
   3. Sometimes
   4. Rarely
   5. Never
9. How would you rate your fatigue on average?
   1. Very severe
   2. Severe
   3. Moderate
   4. Mild
   5. None
10. On a scale of 0 to 10 (where 0 is No Pain, and 10 is Worst Pain Imaginable) how would you rate your pain on average?

## Cluster 4

During the past 3 months, about how often has this happened to you? (Never, Once or a few times, Once or twice a week, Daily or almost daily)

1. I have been unhappy because of my drinking.
2. Because of my drinking, I have not eaten properly.
3. I have failed to do what is expected of me because of my drinking.
4. I have felt guilty or ashamed because of my drinking.
5. I have taken foolish risks when I have been drinking.
6. When drinking, I have done impulsive things that I regretted later.

Now answer these questions about things that may have happened to you. During the past 3 months, how much has this happened? (Not at all, A little, Somewhat, Very much)

1. My physical health has been harmed by my drinking.
2. I have had money problems because of my drinking.
3. My physical appearance has been harmed by my drinking.
4. My family has been hurt by my drinking.
5. A friendship or close relationship has been damaged by my drinking.
6. My drinking has gotten in the way of my growth as a person.
7. My drinking has damaged my social life, popularity, or reputation.
8. I have spent too much or lost a lot of money because of my drinking.
9. I have had an accident while drinking or intoxicated.
   1. No
   2. Almost
   3. Yes, once
   4. Yes, more than once
10. Has this happened to you during the past 3 months: I have had an injury while drinking or intoxicated. (Please include any injuries you may have experienced, even those that were not your fault and those that resulted from the accidents you have already reported):
    1. No
    2. Almost
    3. Yes, once
    4. Yes, more than once
11. During the past 3 months, how many visits have you made to the emergency room or urgent care treatment facility for health treatment?
