# Supplementary material for: The effect of question order on outcomes in the orbital core outcome set for alcohol brief interventions among online help-seekers (QOBCOS): Findings from a randomised factorial trial
Source: Digit Health. 2023 Feb 12;9:20552076231155684. doi: 10.1177/20552076231155684 (PMC9926362; doi:10.1177/20552076231155684)
Supplement: sj-docx-2-dhj-10.1177_20552076231155684 - Supplemental material for The effect of question order on outcomes in the orbital core outcome set for alcohol brief interventions among online help-seekers (QOBCOS): Findings from a randomised factorial trial [file sj-docx-2-dhj-10.1177_20552076231155684.docx]

# Appendix B – Websites referred to post survey completion

After completing the survey, participants were recommended three websites where they could read more about alcohol and health. The websites were:

NIAAA Rethinking Drinking (<https://www.rethinkingdrinking.niaaa.nih.gov/>)

Alcohol Change UK (<https://alcoholchange.org.uk/>)

1177 Vårdguiden (Sweden) (<https://www.1177.se/Ostergotland/liv--halsa/tobak-och-alkohol/sa-kan-du-andra-dina-alkoholvanor/>)
