# Supplementary material for: The effect of question order on outcomes in the orbital core outcome set for alcohol brief interventions among online help-seekers (QOBCOS): Findings from a randomised factorial trial
Source: Digit Health. 2023 Feb 12;9:20552076231155684. doi: 10.1177/20552076231155684 (PMC9926362; doi:10.1177/20552076231155684)
Supplement: sj-docx-3-dhj-10.1177_20552076231155684 - Supplemental material for The effect of question order on outcomes in the orbital core outcome set for alcohol brief interventions among online help-seekers (QOBCOS): Findings from a randomised factorial trial [file sj-docx-3-dhj-10.1177_20552076231155684.docx]

# Appendix C – Futility and harm criteria evaluation

This appendix contains the final evaluation of the target criteria used to guide the decision to stop recruitment. For each regression coefficient representing an order effect, both effect and futility target criteria were evaluated (see Sample Size in the main manuscript for details). In Table 1, the posterior marginal probability of effect and futility is presented, together with a determination of which target was sufficiently satisfied or ambiguous. As can be seen, 6 of the 30 order effects were ambiguous (neither target sufficiently satisfied). However, it was deemed unjustifiable to continue recruiting participants with the purpose of ensuring the targets be satisfied for these six effect estimates. In particular since they were likely to be small in size or that the futility criteria was most likely to be satisfied.

Table 1 - Final evaluation of target criteria

|  | **Posterior marginal probability of:** | |  |
| --- | --- | --- | --- |
|  | **Effect (target 97.5%)** | **Futility (target 95.0%)** | **Sufficiently satisfied** |
| **AUDIT-C Total** | | | |
| Cluster 2 | 97.0% | 11.6% | Effect |
| Cluster 3 | 98.8% | 5.6% | Effect |
| Cluster 4 | > 99.9% | < 0.1% | Effect |
| **AUDIT 1** | | | |
| Cluster 2 | 99.9% | 14.3% | Effect |
| Cluster 3 | 96.1% | 54.4% | Effect |
| Cluster 4 | > 99.9% | < 0.1% | Effect |
| **AUDIT 2** | | | |
| Cluster 2 | 57.2% | 94.5% | Futility |
| Cluster 3 | 98.7% | 37.4% | Effect |
| Cluster 4 | > 99.9% | 0.1% | Effect |
| **AUDIT 3** | | | |
| Cluster 2 | 95.7% | 54.6% | Effect |
| Cluster 3 | 98.3% | 38.2% | Effect |
| Cluster 4 | > 99.9% | 7.2% | Effect |
| **Hazardous and harmful drinking** | | | |
| Cluster 2 | 93.5% | 79.5% | Effect |
| Cluster 3 | 76.2% | 94.8% | Futility |
| Cluster 4 | > 99.9% | 9.5% | Effect |
| **Past week’s consumption** | | | |
| Cluster 1 | 98.7% | 98.3% | Effect |
| Cluster 3 | 52.7% | > 99.9% | Futility |
| Cluster 4 | 54.1% | > 99.9% | Futility |
| **PROMIS Global 10** | | | |
| Cluster 1 | 82.3% | 17.6% | Ambiguous |
| Cluster 2 | 62.8% | 25.1% | Ambiguous |
| Cluster 4 | 99.9% | < 0.1% | Effect |
| **SIP** | | | |
| Cluster 1 | 99.8% | 0.2% | Effect |
| Cluster 2 | 79.2% | 14.7% | Ambiguous |
| Cluster 3 | 99.8% | 0.2% | Effect |
| **Injury** | | | |
| Cluster 1 | 88.0% | 93.4% | Futility |
| Cluster 2 | 73.2% | 97.9% | Futility |
| Cluster 3 | 87.8% | 92.8% | Futility |
| **Emergency health care visits** | | | |
| Cluster 1 | 61.4% | 85.9% | Ambiguous |
| Cluster 2 | 87.4% | 65.5% | Ambiguous |
| Cluster 3 | 70.6% | 79.9% | Ambiguous |
